# Determining the relationship between the knowledge on self-management and levels of asthma control among adult asthmatic patients: a cross-sectional study

**DOI:** 10.25122/jml-2022-0333

**Published:** 2023-03

**Authors:** Muhammad Thesa Ghozali, Ulfah Aulia Urrohmah

**Affiliations:** 1 Department of Pharmaceutical Management, School of Pharmacy, Faculty of Medicine and Health Sciences, Universitas Muhammadiyah Yogyakarta, Yogyakarta, Indonesia

**Keywords:** asthma, asthma control, education, knowledge, self-management

## Abstract

The primary aim of asthma management is to improve the patient's quality of life and keep the symptoms under control, therefore improving the patient's daily activities. Asthmatic patients who know and understand how to control their symptoms could be able to prevent further attacks. Many previous studies have shown the role of patient knowledge regarding asthma self-management in improving asthma control. It was why this study mainly aimed to determine the relationship between patient knowledge of self-management and asthma control levels among adult asthmatic patients in the rural community. The design used analytical observation with a cross-sectional approach to collect data. It involved 100 asthmatic outpatients from two private hospitals owned by the Muhammadiyah Society in the Special Region of Yogyakarta, Indonesia, from February to August 2022. Most participants had a low level of knowledge (66%;n=66), followed by a good level of knowledge (34%;n=34). Regarding the levels of asthma control, it could be confirmed that 61% (n=61) of study participants had uncontrolled asthma, followed by partially controlled (35%;n=35), and controlled (4%;n=4). In terms of the relationship between two variables (patient knowledge of self-management and asthma control levels), the results of Pearson Chi-Square showed a p-value of 0.001, highlighting a relationship between the patient's knowledge of self-management and levels of asthma control. This study concluded that there was a strong relationship between knowledge regarding asthma self-management and asthma control levels.

## INTRODUCTION

Asthma is a major health problem in developed and developing countries. According to the data from the 2022 Global Initiative for Asthma (GINA) report, the prevalence of asthma in various countries was between 1 to 18%, and it was estimated that more than 300 million people worldwide suffer from this respiratory condition [1]. Meanwhile, according to the World Health Organization (WHO), it was estimated that more than 235 million people worldwide have asthma and are underdiagnosed, with a mortality rate of more than 80% in developing countries, mainly due to poverty, lack of education, knowledge, and treatment facilities [2,3].

Based on data from the Basic Health Research of Indonesia in 2013, asthma incidence reached 4.5%. According to the Indonesian Ministry of Health, in 2011, the disease was included in the top ten causes of morbidity and mortality [3]. When not appropriately controlled, according to a scoping review, the death rate is expected to increase by 20% in the following ten years [4]. Controlled asthma is strongly influenced by the patient’s knowledge of the disease history. Furthermore, clinical reports and observational studies show that asthma degree and psychological factors can also directly affect this disease [5,6]. Knowledge of the triggering factors for asthma, such as emotion, was proven to affect lung function directly. Based on observations using pulmonary function monitoring per person, there is also a relationship between the patient’s mood or emotion on the lung function and the lack of patient knowledge while considering asthma is incurable [7]. Moreover, there is a lack of effort to prevent asthma attacks at home and avoid allergens, resulting in recurrence in asthmatic patients [8].

Patients can use the Asthma General Knowledge Questionnaire (AGKQ) and the asthma control level with the Asthma Control Test (ACT) questionnaire to know or determine the relationship between the general knowledge level and the quality of asthma control [9]. Based on the research carried out, good asthma knowledge provides excellent control over its expression. Knowledge of asthma potential triggers is very influential on asthma attacks and the ability to control them. Negative life experiences have been shown to have a substantial effect. This research analyzed the relationship between asthma knowledge and control levels in rural areas, particularly in the Special Region of Yogyakarta, Indonesia. However, the conclusions of this study should be further researched on larger populations, especially for asthmatic patients in rural areas.

## MATERIAL AND METHODS

### Study design and participants

The design of this study was based on analytical observation with a cross-sectional approach to collect data. It involved 100 asthmatic outpatients from two private hospitals in the Special Region of Yogyakarta, Indonesia, with data collected from February to August 2022. Convenience sampling was used. The inclusion criteria were (a) Individuals diagnosed with asthma by a general practitioner or pulmonary specialist; and (b) willingness and ability to complete a series of data collection procedures. Meanwhile, exclusion criteria were: (a) Individuals with other severe lung diseases such as pulmonary tuberculosis, pneumonia, lung cancer, emphysema, and others; (b) people who did not finish and completely answered all the question items on the questionnaire; and (c) individuals who withdrew from the study.

### Research instruments

The research instrument was a questionnaire. The Asthma General Knowledge Questionnaire (AGKQ) [10] and the Asthma Control Test (ACT) questionnaire were used to measure the asthma control level [11].

#### Asthma General Knowledge Questionnaire (AGKQ)

General knowledge of asthma patients describes all information on the disease after sensing sight, hearing, or feeling, and the test equipment is generally based on the AGKQ questionnaire. Meanwhile, the questionnaire can be accepted validly and reliably for the testing process. It has also proved to be a valuable research tool for determining asthma knowledge levels in educational or clinical interventions. The questionnaire metrics are divided into (a) Low levels - when the correct answer is less than 60%, and (b) High levels - when the correct answer is greater than or equal to 60%.

#### Asthma control test

The Asthma Control Test (ACT) questionnaire assessed the asthma control level. ACT is a tool that can be used to evaluate asthma control in patients, and its use is recommended. This tool is straightforward because it contains five questions the patient should fill in, and a score of 1 to 5 is given for each answer. The maximum score is 25 with the following divisions: (a) Not controlled (score ≤19), (b) Partially controlled (score 20-24), and (c) controlled (score 25). In addition, it can improve the communication quality between doctors and patients because the questions on the ACT are clear and consistent. Therefore, patients are more open and can answer questions honestly.

### Research procedures

The data were collected through questionnaires applied to all the selected participants. After receiving a brief explanation of the purpose of this study, the participants were asked to fill out the Asthma General Knowledge Questionnaire and Asthma Control Test questionnaires. The data were then processed statistically to determine the patient’s asthma knowledge, the control quality level in the last four weeks, and the relationship between the two variables.

### Data analysis

The data were recorded and further processed for statistical tests using the IBM SPSS Statistics 26 version software and analyzed using univariate and bivariate analysis. The univariate analysis aimed to obtain an overview of each variable distribution, and the bivariate analysis to determine the relationship between knowledge of patient self-management and levels of asthma control. Bivariate analysis was carried out with the Chi-Square significance test to obtain the relationship between independent and dependent variables.

## RESULTS

### Characteristics of respondents

The convenience sampling resulted in 112 prospective respondents. However, 7 study participants did not complete the questionnaire for unclear reasons, and 5 withdrew since they could not be contacted. According to Table 1, the study was dominated by male respondents (66%;n=67), aged between 15–20 years (61%;n=61), undergraduate (77%;n=71), and working as a student (69%;n=69). Regarding patient knowledge of self-management, more than half of the participants had a low level (66%;n=66). Moreover, in terms of asthma control levels, most had uncontrolled asthma (61%;n=61).

**Table 1:** Demographic characteristics of study participants (n=100).

Demographics	n (%)
**Gender**	
Male	67 (67%)
Female	33 (33%)
**Age (years old)**	
15–20	61 (61%)
21–25	39 (39%)
**Levels of education**	
Undergraduate	77 (77%)
Graduate	23 (23%)
**Occupation**	
Student	69 (69%)
Entrepreneur	14 (9%)
Private employee	11 (11%)
Housewife	4 (4%)
Government employee	1 (1%)
Unemployed	1 (1%)
**Knowledge on self-management**	
High	34 (34%)
Low	66 (66%)
**Levels of asthma control**	
Controlled	4 (4%)
Partially controlled	35 (35%)
Uncontrolled	61 (61%)

### Participant’s characteristics based on the knowledge of self-management

The asthma knowledge of each respondent was different. Therefore, in this study, a univariate analysis was carried out on the knowledge level. The characteristics distribution of research respondents based on the asthma knowledge level can be seen in Table 2. The study’s findings showed that 66 and 34 respondents had low and high levels of knowledge, 66% and 34%, respectively.

**Table 2:** The descriptive analysis result of the knowledge on self-management (n=100).

Asthma knowledge level	N (%)
**High**	34 (34%)
**Low**	66 (66%)

### Characteristics of research respondents based on asthma control level

The asthma control level of each respondent has a different value. Therefore, a univariate analysis was also implemented on the control level of the respondents. The characteristics distribution based on the respondents’ asthma knowledge level is showcased in Table 3. From the Table, 4% (n=4), 35% (n=35), and 61% (n=61) of study participants have fully controlled, partially controlled, and uncontrolled asthma, respectively.

**Table 3:** The results of descriptive analysis of the level of asthma control.

Asthma knowledge level	n(%)
**Controlled**	4 (4%)
**Partially controlled**	35 (35%)
**Uncontrolled**	61 (61%)

### The relationship between knowledge and asthma control levels

The relationship between asthma knowledge and control level can be seen in Table 4. Participants who had high knowledge of asthma self-management with fully controlled, partially controlled, and uncontrolled levels of asthma knowledge were 4, 27, and 3 (34 in total). Meanwhile, participants who had low knowledge of asthma self-management with fully controlled, partially controlled, and uncontrolled were 0, 8, and 58 (66 in total).

**Table 4:** The knowledge on self-management and levels of asthma control.

		Control level			Total
		Controlled n(%)	Partially n(%)	Uncontrolled n(%)	
**Knowledge**	High	4	27	3	34
	Low	0	8	58	66
**Total**		4	35	61	100

Bivariate analysis of the relationship between asthma knowledge and control level in respondents used the Chi-Square significance test to determine the relationship between the dependent and independent variables. The results are seen in Table 5. Because the p-value of the test was less than 0.05, it can be concluded that “there is a relationship between patient’s knowledge on self-management and levels of asthma control”.

**Table 5:** The results of pearson Chi-Square of the level of asthma control.

**Pearson Chi-Square**	0.001

## DISCUSSION

Efforts to prevent recurrence depend on the patient’s asthma knowledge. Patients should be educated about the disease to recognize the triggering factors for attacks and understand the prevention and treatment. This strategy reduces the symptoms’ frequency, impact on lifestyle, and recurrence. Good knowledge helps patients prevent a recurrence of asthma attacks [12,13].

Patients with recurrent asthma, according to Sánchez et al. (2014), should undergo examinations to identify substances that trigger the disease. Furthermore, a patient history should be conducted to identify asthma triggers when there is a recurrence pattern. In terms of psychosociology, knowing the patient’s ability to handle asthma and adapt to the disease is very important. Patients who feel capable of dealing with asthma are usually more obedient to therapy. A high-stress lifestyle can worsen asthma, and patients who have just been diagnosed need to identify objects and environments triggering recurrence. In addition, an allergy test can also be performed on the skin [14].

### Respondents’ criteria based on asthma knowledge level

The levels of knowledge on asthma self-management were assessed using the Asthma General Knowledge Questionnaire (AGKQ). The results were obtained from 100 study participants with asthma who generally had a low level of knowledge (66%). The results were similar to previous studies, one revealing 58 patients with a low level of knowledge and another 16 people (53.3%) with a low level of asthma knowledge [15, 16]. These results can be explained by the factors that affected the participant’s asthma knowledge level [17].

Factors influencing knowledge vary from person to person but may include education, information/social media, social culture, environment, experience, and age [17]. Education is the most dominant factor in improving an individual’s knowledge. Culture affects the level as new information will be filtered and adapted to the existing culture and religion. Meanwhile, experience highly relates to the individual’s age and education. The higher the education, the wider the experience. Education affects a person’s attitudes, actions, and thoughts, with higher education positively influencing behavior [18]. The mindset that results from the knowledge gained will impact actions, attitudes, and the environment. It shows that people with higher education tend to be more concerned about their health and make more efforts to improve their health status by going to facilities for treatment. Asthma needs attention since it can reduce productivity and increase the economic burden. Knowledge about the disease needs to be available to the general public to minimize the triggering factors for the patients.

### Respondents’ criteria based on asthma control level

The asthma control level was obtained based on the results of the Asthma Control Test (ACT) questionnaire. The highest ACT score, when considering the number of patients, was the uncontrolled asthma control level (n=61, 61%), partially controlled (35, 35%), and the lowest score was the controlled asthma level (n=4, 4%). It was in line with a previous study, with an uncontrolled asthma prevalence of 81 (73.7%) followed by controlled asthma 26 (24.3%) [19]. According to the study, the high prevalence of uncontrolled asthma was influenced by various factors, including age, gender, genes, comorbid diseases, smoking, use of corticosteroid drugs, weight, poor treatment habits, and knowledge level. These factors can affect the originally good level due to aging and excess body weight. Therefore, there are many considerations for re-controlling [19].

### The relationship between knowledge levels of asthma and asthma control levels

Based on the Chi-Square statistical test results by looking at the Asymp value. Sig. (2-sided) or p-value in the Pearson Chi-Square test was 0.001. Furthermore, when the Chi-Square test results have a p-value of 0.001 <0.005, revealing a relationship between the patient’s knowledge of self-management and levels of asthma control. The findings followed a previous study, which confirmed a strong relationship between the two variables [20]. A low level of knowledge is a factor that affects the asthma severity in patients and affects the control status to become uncontrolled.

Similar results were revealed by other research, which stated that good knowledge about asthma could increase behavior control by 6,682 times [21]. A systematic review and meta-analysis also confirmed a significant relationship between the knowledge of self-management and levels of asthma control [22]. Unfortunately, the results differ from this previous study [21], which did not find a significant relationship between these variables, possibly due to confounding variables that were not considered and might affect the results [23].

Knowledge about asthma is defined as the respondent's ability to correctly answer various questions guided by the Asthma General Knowledge Questionnaire (AGKQ). This study indicates that respondents have a low level of knowledge related to asthma (n=66;66%). It can occur due to various factors, such as asthmatics' lack of awareness of the disease. Moreover, health workers' lack of socialization and communication in providing preventive information to the community can cause reduced knowledge, highlighting the importance of an active role of the medical personnel.

The common knowledge of asthmatics about the disease, if not delivered properly, tends to bring patients to be categorized into uncontrolled asthma. It can be seen in this study with uncontrolled asthma levels reaching 61 respondents (61%). The reason behind this can be a lack of awareness, knowledge, desire, and asthmatics' proper behavior to control the disease. Another research stated that high knowledge of asthmatics would impact better self-management of patients; hence controlled asthma can be achieved. Consequently, self-management and good medical therapy are needed [24].

Self-management of asthma refers to the several ways to monitor and control symptoms. Training patients should be part of routine clinical services and address relevant cognitive variables such as knowledge, attitudes, and self-management efficacy. Asthma knowledge includes recognizing triggers, understanding the patient's role in care and treatment, and developing plans for critical situations [25].

Good education about self-management can reduce asthma morbidity in adults. It allows patients to increase their general knowledge and understanding patterns, improving their skills, compliance, and self-management. Providing asthma treatment education to people improves disease control. Knowledge about asthma significantly contributes to self-management, adherence to treatment plans, and control of environmental factors that can trigger the disease [26,27].

Cognitive characteristics of asthmatics will impact self-management, ultimately affecting the patient's status. An increase in knowledge, attitudes, and personal abilities in self-management will provide benefits in achieving controlled asthma status and better life quality for patients.

## CONCLUSION

There was a strong relationship between knowledge of self-management and levels of asthma control. Based on the findings of this study, it is highly suggested that education is necessary to improve patients' knowledge about asthma and its control level.

## Data Availability

Further data is available from the corresponding author on reasonable request.
